# Checklist of British and Irish Hymenoptera - Ceraphronoidea

**DOI:** 10.3897/BDJ.2.e1167

**Published:** 2014-08-27

**Authors:** Gavin R. Broad, Laurence Livermore

**Affiliations:** † The Natural History Museum, London, London, United Kingdom; ‡ The Natural History Museum, London, United Kingdom

## Introduction

This checklist comprises parts of the larger checklist of the Hymenoptera of the British Isles; the terms of reference and methodology are explained in Broad (2014). Country level distribution records, as reported here, are mainly taken from the collections of the Natural History Museum, London (NHM), with additional sources cited. The Ceraphronoidea comprises two extant families, the Ceraphronidae and Megaspilidae, represented by 28 and 64 valid species in the British Isles. Representatives of the British families and subfamilies of Ceraphronoidea are shown in Fig. 1a, c, d. There has been very little recent work on the British and Irish Ceraphronoidea and it is generally a rather neglected group of parasitoids. The genus *Dendrocerus* is an exception: several species are frequently reared as hyperparasitoids of aphids or from other aphid-associated insects and their taxonomy has been revised (Fergusson 1980). Much remains to be discovered about the biology and taxonomy of ceraphronoids. Whilst at least some megaspilids are known to be ectoparasitoid idiobionts (Haviland 1920), including *Dendrocerus*, the Ceraphronidae contains ecto- and endoparasitoids; within the same genus (*Aphanogmus*) some species seem to be endoparasitoids (e.g. Parnell 1963) and others ectoparasitoids (Luhman et al. 1999, Evans et al. 2005), with idiobiosis and koinobiosis both reported in the literature. Whilst hosts of both ceraphronids and megaspilids seem to be centred on Diptera there are many exceptions and the range of hosts of the superfamily spans several orders. Nomenclature here follows Johnson and Musetti (2004) a rather full synonymy is given as there is only a very small body of literature dealing with ceraphronoids in a British context, so most names are unfamiliar to most entomologists.

## Checklists

### Superfamily Ceraphronoidea

#### Ceraphronidae

Haliday, 1833

#### Aphanogmus

Thomson, 1858

##### Notes

Information on a few species, including some distribution data, is given by Parr (1960) and Parnell (1963).

#### Aphanogmus abdominalis

(Thomson, 1858)

Calliceras abdominalis Thomson, 1858
Ceraphron pallidiventris (Ashmead, 1893, *Ceraphron*)
Ceraphron cameroni (Kieffer, 1907, *Ceraphron*)
Ceraphron microneurus (Kieffer, 1907, *Ceraphron*) preocc.
Calliceras violae (Novitzky, 1954, *Calliceras*)

##### Distribution

England

##### Notes

See Buhl (2009).

#### Aphanogmus bicolor

Ashmead, 1893

Ceraphron aviger (Kieffer, 1907, *Ceraphron*)
Ceraphron reitteri (Kieffer, 1907, *Ceraphron*)
Calliceras humicola (Novitzky, 1954, *Calliceras*)

#### Aphanogmus compressus

(Ratzeburg, 1852)

Hadroceras compressa Ratzeburg, 1852
Aphanogmus nigrofornicatus Pschorn-Walcher, 1956
Aphanogmus annulicornis Jansson, 1957
Aphanogmus venustus Parr, 1960 synonymy by Dessart (1991)

##### Distribution

England, Ireland

#### Aphanogmus elegantulus

Förster, 1861

##### Distribution

Ireland

##### Notes

Added by Buhl and O'Connor (2012).

#### Aphanogmus fasciipennis

Thomson, 1858

Aphanogmus fusciipennis misspelling

##### Distribution

England

#### Aphanogmus fumipennis

Thomson, 1858

Aphanogmus hyalinipennis Thomson, 1858
Aphanogmus laevis Förster, 1861
Aphanogmus grenadensis Ashmead, 1896
Aphanogmus formicarius Kieffer, 1905
Aphanogmus ?clavatus Kieffer, 1907
Ceraphron armatus (Kieffer, 1907, *Ceraphron*) preocc.
Ceraphron formicarum (Kieffer, 1907, *Ceraphron*)
Ceraphron frenalis (Kieffer, 1907, *Ceraphron*)
Ceraphron oriphilus (Kieffer, 1913, *Ceraphron*)
Ceraphron fuliginosi (Box, 1921, *Ceraphron*)
Calliceras fasciatus (Fouts, 1924, *Calliceras*)
Calliceras borealis (Whittaker, 1930, *Calliceras*)
Allomicrops bemisiae (Ghesquière, 1935, *Allomicrops*)
Ceraphron roberti (Dessart, 1979, *Ceraphron*)

##### Distribution

England, Ireland

#### Aphanogmus furcatus

Kieffer, 1907

#### Aphanogmus microneurus

Kieffer, 1907

Aphanogmus obsoletus Whittaker, 1930
Aphanogmus cylindricornis Parr, 1960 synonymy by Dessart (1965)

##### Distribution

England

#### Aphanogmus nanus

(Nees, 1834)

Calliceras nigriceps (Thomson, 1858, *Calliceras*)
Calliceras pallidus (Thomson, 1858, *Calliceras*)
Ceraphron nigriclavis (Förster, 1861, *Ceraphron*)

##### Distribution

Ireland

##### Notes

Recorded, as new to Ireland, as *Ceraphron nigriceps* (Thomson) by O'Connor (2007).

#### Aphanogmus myrmecobius

Kieffer, 1914

#### Aphanogmus sagena

Johnson & Musetti, 2004

Aphanogmus reticulatus Parr, 1960 preocc.

##### Distribution

England

#### Aphanogmus steinitzi

Priesner, 1936

##### Distribution

England

#### Aphanogmus tenuicornis

Thomson, 1858

Ceraphron ultimus (Dalla Torre, 1890, *Ceraphron*)

##### Distribution

Ireland

#### Aphanogmus terminalis

Förster, 1861

##### Distribution

Ireland

##### Notes

Added by Buhl and O'Connor (2011).

#### Aphanogmus vicinus

Förster, 1861

##### Distribution

England, Ireland

##### Notes

Added by Buhl and O'Connor (2012).

#### Ceraphron

Jurine, 1807

CALLICERAS Nees, 1834
HADROCERAS Förster, 1840
TOMOLIGON Rondani, 1877
MEGASPILIDEA Ashmead, 1888
NEOCERAPHRON Ashmead, 1893
PRISTOMICROPS Kieffer, 1906
CERATOPHRON Schulz, 1906 emendation
ALLOMICROPS Kieffer, 1914
EULAGYNODES Girault, 1917
CERANOGMUS Risbec, 1953
LARSOCERAPHRON Dessart, 1981
OKTOCERAPHRON Dessart & Cancemi, 1987

#### Ceraphron bispinosus

(Nees, 1834)

Calliceras bispinosa Nees, 1834
Ceraphron striatus Förster, 1861
Ceraphron crassicornis Harrington, 1899
Ceraphron opacus Kieffer, 1905
Calliceras bohemani (Kieffer, 1914, *Calliceras*)
Calliceras septentrionalis (Kieffer, 1914, *Calliceras*)

#### Ceraphron flaviventris

Kieffer, 1907

#### Ceraphron myrmecophilus

Kieffer, 1913

#### Ceraphron myrmicarum

Kieffer, 1913

#### Ceraphron nigraticeps

Kieffer, 1907

##### Distribution

England

#### Ceraphron nigrelliceps

Kieffer, 1907

#### Ceraphron pallipes

(Thomson, 1858)

Calliceras pallipes Thomson, 1858
Ceraphron pallidipes misspelling
Ceraphron globuliflagellaris Szabó, 1979

#### Ceraphron scoticus

Kieffer, 1907

##### Distribution

England, Scotland

#### Ceraphron testaceipes

Kieffer, 1904

Ceraphron bruneus Kieffer, 1904
Ceraphron spinifer Kieffer, 1907
Ceraphron spinifex misspelling

##### Distribution

England

#### Ceraphron trissacantha

Kieffer, 1907

##### Distribution

Ireland

##### Notes

Added by Buhl and O'Connor (2011).

#### Synaris

Förster, 1878

SINARIS misspelling

#### Synaris britannica

Szelényi, 1936

#### Synaris planifrons

Kieffer, 1907

#### Synaris pulla

Förster, 1878

##### Distribution

England

#### Megaspilidae

Ashmead, 1893

#### Lagynodinae

Masner & Dessart, 1967

#### Lagynodes

Förster, 1840

LAGNYODES misspelling
MICROPS Haliday, 1833 preocc.
MICROPS Marshall, 1874
PLASTOMICROPS Kieffer, 1906

#### Lagynodes pallidus

(Boheman, 1832)

Ceraphron pallidus Boheman, 1832
Microps rubi (Haliday, 1833, *Microps*)
Ceraphron pallidus (Zetterstedt, 1840, *Ceraphron*) preocc.
Hadrocerus spinosa (Förster, 1840, *Hadrocerus*)
Lagynodes rufus Förster, 1840
Lagynodes rufescens Ruthe, 1859
Triogmus furcifer (Marshall, 1874, *Triogmus*)
Lagynodes minutus Ashmead, 1893
Lagynodes nitidiceps Kieffer, 1906
Lagynodes crassicornis Kieffer, 1906
Lagynodes niger Kieffer, 1906
Lagynodes aterior Box, 1921

##### Distribution

England, Wales, Ireland

#### Lagynodes thoracicus

Kieffer, 1906

Lagynodes janssoni Maumené-Burtel, 1957

#### Megaspilinae

Ashmead, 1893

#### Conostigmus

Dahlbom, 1858

DICHOGMUS Thomson, 1858
EUMEGASPILUS Ashmead, 1888
EUMEGALOSPILUS Schulz, 1906 invalid emendation
CONOSTIGMOIDES Dodd, 1914
ECNOMOTHORAX Dessart & Masner, 1965
DOLICHOCERAPHRON Hellén, 1966
SZELENYIDES Dessart, 1974

#### Conostigmus abdominalis

(Boheman, 1832)

Ceraphron abdominalis Boheman, 1832
Ceraphron tenuicornis (Boheman, 1832, *Ceraphron*)
Conostigmus testacea Kieffer, 1907
Conostigmus divisifrons Kieffer, 1907
Conostigmus foveatifrons Kieffer, 1907
Conostigmus pilosiceps Szabó, 1979
Conostigmus curvilineaticeps Szabó, 1979

##### Distribution

England

#### Conostigmus alutaceus

(Thomson, 1858)

Megaspilus alutaceus Thomson, 1858

#### Conostigmus apteryx

Kieffer, 1909

Conostigmus apterus Kieffer, 1907 preocc.

#### Conostigmus atelopterus

(Marshall, 1868)

Megaspilus atelopterus Marshall, 1868

#### Conostigmus basalis

Kieffer, 1907

#### Conostigmus borealis

(Thomson, 1858)

Megaspilus borealis Thomson, 1858

#### Conostigmus brachypterus

(Thomson, 1858)

Megaspilus brachypterus Thomson, 1858
Ceraphron subapterus (Zetterstedt, 1840, *Ceraphron*) preocc.

#### Conostigmus britannicus

Kieffer, 1907

Conostigmus cameroni Kieffer, 1914

#### Conostigmus carpentieri

Kieffer, 1907

##### Distribution

England, Ireland

#### Conostigmus clavicornis

Kieffer, 1907

#### Conostigmus crassicornis

(Boheman, 1832)

Ceraphron crassicornis Boheman, 1832
Megaspilus validicornis (Thomson, 1858, *Megaspilus*)
Conostigmus flavopunctatus Kieffer, 1907
Conostigmus macrocerus Kieffer, 1907
Conostigmus alpicola Kieffer, 1907
Conostigmus subclavatus Kieffer, 1907
Conostigmus apsteini Kieffer, 1914

##### Distribution

England, Ireland

#### Conostigmus cursitans

(Nees, 1834)

Calliceras cursitans Nees, 1834
Conostigmus leptothorax Kieffer, 1907
Conostigmus micans Kieffer, 1907
Conostigmus subalatus Kieffer, 1907

##### Distribution

England

#### Conostigmus curtipennis

Kieffer, 1907

#### Conostigmus cylindricus

Kieffer, 1907

Conostigmus geniculatus Kieffer, 1907
Dichogmus nigriceps (Kieffer, 1907, *Dichogmus*)
Conostigmus globiceps Hellén, 1966

##### Distribution

England, Ireland

#### Conostigmus fasciatipennis

Kieffer, 1907

##### Distribution

England

#### Conostigmus formiceti

(Erichson, 1844)

Megaspilus wasmanni (Kieffer, 1904, *Megaspilus*)
Megaspilus antennalis (Kieffer, 1904, *Megaspilus*)
Megaspilus testaceipes (Kieffer, 1904, *Megaspilus*)
Megaspilus ?lasiophilus (Kieffer, 1905, *Megaspilus*)
Conostigmus tricolor Kieffer, 1907
Conostigmus myrmecobius Kieffer, 1913
Conostigmus formicarum Kieffer, 1914
Conostigmus nidorum Kieffer, 1914

##### Distribution

England

#### Conostigmus frontalis

(Thomson, 1858)

Megaspilus crassinervis (Kieffer, 1904, *Megaspilus*)
Conostigmus subsulcatus Kieffer, 1907

##### Distribution

England

#### Conostigmus fuscipes

(Nees, 1834)

#### Conostigmus halteratus

(Boheman, 1832)

Ceraphron halteratus Boheman, 1832
Ceraphron longicornis (Boheman, 1832, *Ceraphron*)
Calliceras brevipennis (Nees, 1834, *Calliceras*)
Megaspilus punctulatus (Cameron, 1881, *Megaspilus*)
Megaspilus cursor (Kieffer, 1904, *Megaspilus*)
Megaspilus clavatipennis clavatipennis (Kieffer, 1905, *Megaspilus*)
Conostigmus rhopalophorus Kieffer, 1907
Conostigmus lineatifrons Kieffer, 1907
Conostigmus punctatifrons Kieffer, 1907

##### Distribution

England, Scotland

#### Conostigmus humilis

Kieffer, 1907

##### Distribution

England

#### Conostigmus inconstans

Kieffer, 1907

Conostigmus angusticeps Kieffer, 1907
Conostigmus pennatus Kieffer, 1907

#### Conostigmus innotatus

Kieffer, 1907

#### Conostigmus lativentris

(Thomson, 1858)

Megaspilus lativentris Thomson, 1858
Conostigmus halteriger Kieffer, 1907
Conostigmus scabriventris Kieffer, 1907
Conostigmus holoceps Szabó, 1979

##### Distribution

England

#### Conostigmus levifrons

Kieffer, 1907

Conostigmus semipunctatus Szabó, 1979
Conostigmus szelenyii Szabó, 1979

##### Distribution

England

#### Conostigmus linearis

Hellén, 1966

##### Distribution

England

##### Notes

Added by Dessart (1996).

#### Conostigmus lucidus

Kieffer, 1907

##### Distribution

England, Ireland

#### Conostigmus melanocephalus

(Boheman, 1832)

Ceraphron melanocephalus Boheman, 1832
Calliceras thoracicus (Nees, 1834, *Calliceras*)
Conostigmus allotropus Kieffer, 1907
Conostigmus micromma Kieffer, 1907
Conostigmus signatifrons Kieffer, 1917

#### Conostigmus mullensis

(Cameron, 1881)

Megaspilus mullensis Cameron, 1881

##### Distribution

Scotland

#### Conostigmus niger

Kieffer, 1907

#### Conostigmus nigriventris

Kieffer, 1907

#### Conostigmus obscurus

(Thomson, 1858)

Megaspilus obscurus Thomson, 1858
Megaspilus arcticus (Thomson, 1858, *Megaspilus*)
Conostigmus syrphorum Kieffer, 1907

#### Conostigmus planifrons

Kieffer, 1907

#### Conostigmus pubescens

(Thomson, 1858)

Ceraphron pubescens Thomson, 1858

##### Distribution

England, Ireland

#### Conostigmus rufescens

Kieffer, 1907

Conostigmus anglicus Kieffer, 1907

##### Distribution

England

#### Conostigmus ruficollis

Kieffer, 1907

Conostigmus globuliceps Szabó, 1979

#### Conostigmus rufipes

(Nees, 1834)

Ceraphron rufipes Nees, 1834
Conostigmus cruciger Szabó, 1979
Conostigmus misinus Szabó, 1979

##### Distribution

England

#### Conostigmus rutilus

Kieffer, 1907

#### Conostigmus speculiger

Dessart, 1974

#### Conostigmus subclavicornis

Kieffer, 1907

#### Conostigmus subfilicornis

Kieffer, 1907

##### Distribution

England

#### Conostigmus sulcaticeps

Kieffer, 1907

#### Conostigmus triangularis

(Thomson, 1858)

Ceraphron triangularis Thomson, 1858
Trichosteresis armatus (Kieffer, 1907, *Trichosteresis*)
Conostigmus marshalli Kieffer, 1907
Conostigmus forticornis Kieffer, 1907
Conostigmus ater Fouts, 1926
Conostigmus zagloui Kamal, 1926
Conostigmus moczari Szabó, 1979

##### Distribution

England

#### Conostigmus tristriatus

Kieffer, 1907

#### Conostigmus versicolor

Kieffer, 1907

##### Distribution

England, Wales, Ireland

#### Creator

Alekseev, 1980

#### Creator spissicornis

(Hellén, 1966)

Lygocerus spissicornis Hellén, 1966

##### Distribution

England

##### Notes

Added by Fergusson (1980). Treated as a species of *Dendrocerus* by Fergusson (1980).

#### Dendrocerus

Ratzeburg, 1852

LYGOCERUS Förster, 1856
MACROSTIGMA Rondani, 1877
ATRITOMUS Förster, 1878 preocc.
PRODENDROCERUS Kieffer, 1907
ATRITOMELLUS Kieffer, 1914
NEOLYGOCERUS Ishii, 1951
BASOKO Risbec, 1958

##### Notes

Syonymic and distributional data taken from Fergusson (1980).

#### Dendrocerus aphidum

(Rondani, 1877)

Macrostigma aphidum Rondani, 1877
Ceraphron rufipes (Thomson, 1858, *Ceraphron*) preocc.
Macrostigma aphidum (Rondani, 1877, *Macrostigma*)
Lygocerus koebelei (Ashmead, 1904, *Lygocerus*)
Lygocerus subquadratus (Kieffer, 1907, *Lygocerus*)
Lygocerus fusciventris (Kieffer, 1907, *Lygocerus*)
Lygocerus frenalis (Kieffer, 1907, *Lygocerus*)
Lygocerus breadalbimensis (Kieffer, 1907, *Lygocerus*)
Lygocerus bicolor (Kieffer, 1907, *Lygocerus*)
Lygocerus fuscipennis (Kieffer, 1907, *Lygocerus*)
Lygocerus ?neglectus (Kieffer, 1907, *Lygocerus*)
Dendrocerus attentus Muesebeck, 1959
Dendrocerus lundensis Dessart, 1966

##### Distribution

England, Scotland, Wales, Ireland

#### Dendrocerus bifoveatus

(Kieffer, 1907)

Lygocerus bifoveatus Kieffer, 1907
Lygocerus sordidipes (Kieffer, 1907, *Lygocerus*)

##### Distribution

England, Scotland

#### Dendrocerus carpenteri

(Curtis, 1829)

Ceraphron carpenteri Curtis, 1829
Ceraphron crispus (Curtis, 1829, *Ceraphron*) nom. nud.
Ceraphron elegans (Curtis, 1829, *Ceraphron*) nom. nud.
Ceraphron fuscipes (Ratzeburg, 1852, *Ceraphron*) preocc.
Ceraphron hyalinatus (Thomson, 1858, *Ceraphron*)
Megaspilus niger (Howard, 1890, *Megaspilus*)
Trichosteresis proximus (Kieffer, 1907, *Trichosteresis*)
Trichosteresis punctatipennis (Kieffer, 1907, *Trichosteresis*)
Lygocerus rufiventris (Kieffer, 1907, *Lygocerus*) preocc.
Lygocerus campestris (Kieffer, 1907, *Lygocerus*)
Lygocerus aphidivorus (Kieffer, 1907, *Lygocerus*)
Lygocerus testaceimanus (Kieffer, 1907, *Lygocerus*)
Lygocerus aphidum (Kieffer, 1907, *Lygocerus*) preocc.
Lygocerus giraudi (Kieffer, 1907, *Lygocerus*)
Lygocerus cameroni (Kieffer, 1907, *Lygocerus*)
Lygocerus thomsoni (Kieffer, 1907, *Lygocerus*)
Lygocerus inquilinus (Kieffer, 1917, *Lygocerus*)
Lygocerus ambianus (Dessart, 1965, *Lygocerus*)
Dendrocerus britannicus Dessart, 1966
Dendrocerus tischbeini Dessart, 1966

##### Distribution

England, Scotland, Wales, Ireland

#### Dendrocerus flavipes

Kieffer, 1907

Dendrocerus fuscipes Kieffer, 1907

##### Distribution

England, Ireland

##### Notes

Added by Fergusson (1980).

#### Dendrocerus ?floridanus

(Ashmead, 1881)

Chirocerus floridanus Ashmead, 1881
Lygocerus semiramosus (Kieffer, 1907, *Lygocerus*)
Lygocerus longispinus (Yasumatsu & Moritsu, 1947, *Lygocerus*)

##### Notes

Provisionally included on the British and Irish list on the basis of Dessart (1978), who synonymised *Lygocerus semiramosus* (described from a British specimen) under *Dendrocerus floridanus*. Although Fergusson (1980) listed *Lygocerus semiramosus* as a junior synonym of *Dendrocerus serricornis*, it was retained as a junior synonym of *Dendrocerus floridanus* by Johnson and Musetti (2004), albeit with the comment that this ‘difference in taxonomic judgement has not yet been resolved’.

#### Dendrocerus halidayi

(Curtis, 1829)

Ceraphron halidayi Curtis, 1829
Dendrocerus lichtensteinii Ratzeburg, 1852
Ceraphron callicerus (Thomson, 1858, *Ceraphron*)
Atritomellus hungaricus (Szabó, 1979, *Atritomellus*)

##### Distribution

England, Ireland

#### Dendrocerus laevis

(Ratzeburg, 1852)

Ceraphron laevis Ratzeburg, 1852
Ceraphron frontalis (Thomson, 1858, *Ceraphron*)
Atritomus coccophagus (Förster, 1878, *Atritomus*)
Atritomellus smirnoffi (Ghesquière, 1960, *Atritomellus*)

##### Distribution

England, Ireland

##### Notes

Added by Fergusson (1980).

#### Dendrocerus laticeps

(Hedicke, 1929)

Atritomellus laticeps Hedicke, 1929
Lygocerus incompletus (Muesebeck, 1959, *Lygocerus*)

##### Distribution

England, Wales

##### Notes

Added by Fergusson (1980).

#### Dendrocerus liebscheri

Dessart, 1972

Ceraphron tenuicornis (Thomson, 1858, *Ceraphron*) preocc.

##### Distribution

England, Wales

##### Notes

Added by Fergusson (1980).

#### Dendrocerus punctipes

(Boheman, 1832)

Ceraphron punctipes Boheman, 1832
Ceraphron parvulus (Wollaston, 1858, *Ceraphron*)
Conostigmus vassae (Szabó, 1979, *Conostigmus*)

##### Distribution

England

##### Notes

Added by Fergusson (1980).

#### Dendrocerus pupparum

(Boheman, 1832)

Ceraphron pupparum Boheman, 1832
Dendrocerus puparum misspelling
Ceraphron ancyloneurus (Ratzeburg, 1844, *Ceraphron*)
Lygocerus syrphidarum (Kieffer, 1907, *Lygocerus*)

##### Distribution

England

#### Dendrocerus ramicornis

(Boheman, 1832)

Ceraphron ramicornis Boheman, 1832
Ceraphron glabriculus (Thomson, 1858, *Ceraphron*)
Lygocerus japonicus (Ashmead, 1904, *Lygocerus*)
Lygocerus ratzeburgi (Ashmead, 1904, *Lygocerus*)

##### Distribution

England, Scotland

#### Dendrocerus rectangularis

(Kieffer, 1907)

Lygocerus rectangularis Kieffer, 1907
Dendrocerus longicornis (Thomson, 1858, Ceraphron) preocc.
Lygocerus pallipes (Kirchner, 1867, *Lygocerus*) nom. nud.
Lygocerus claripennis (Kieffer, 1907, *Lygocerus*)
Lygocerus flavipes (Kieffer, 1907, *Lygocerus*) preocc.
Conostigmus lentus (Kieffer, 1907, *Conostigmus*)
Conostigmus alpestris (Kieffer, 1907, *Conostigmus*)
Conostigmus dubiosus (Kieffer, 1907, *Conostigmus*) synonymy by Dessart (1981)
Dendrocerus navaensis Dessart, 1966
Lygocerus flavus (Hellén, 1966, *Lygocerus*)

##### Distribution

England, Scotland, Wales, Ireland

#### Dendrocerus serricornis

(Boheman, 1832)

Ceraphron serricornis Boheman, 1832
Dendrocerus serraticornis misspelling
Dendrocerus testaceimanus misident.
Ceraphron piceus (Ratzeburg, 1852, *Ceraphron*)
Ceraphron lapponicus (Thomson, 1858, *Ceraphron*)
Lygocerus subramosus (Kieffer, 1907, *Lygocerus*)
Lygocerus pinicola (Muesebeck, 1959, *Lygocerus*)
Atritomellus zetterstedti (Ghesquière, 1960, *Atritomellus*)

##### Distribution

England, Scotland, Ireland

#### Megaspilus

Westwood, 1829

HABROPELTE Thomson, 1858
MEGASPILODES Ashmead, 1888
MEGALOSPILUS Schulz, 1906 emendation

#### Megaspilus dux

(Curtis, 1829)

Ceraphron dux Curtis, 1829
Ceraphron scutellaris (Boheman, 1832, *Ceraphron*)
Ceraphron tibialis (Boheman, 1832, *Ceraphron*)
Ceraphron herculeus (Förster, 1840, *Ceraphron*)
Telenomus stygicus (Provancher, 1887, *Telenomus*)
Megaspilus pleuralis Kieffer, 1907
Megaspilus integrifrons Kieffer, 1907
Megaspilus rufimanus Kieffer, 1907
Megaspilus merceti Kieffer, 1907
Conostigmus fuscicrus (Kieffer, 1907, *Conostigmus*)

##### Distribution

England

#### Megaspilus striolatus

(Thomson, 1858)

Habropelte striolata Thomson, 1858
Megaspilus hispanicus Kieffer, 1907
Megaspilus flavimanus Kieffer, 1907
Megaspilus sculpturatus Kieffer, 1907

#### Trichosteresis

Förster, 1856

THLIBONEURA Thomson, 1858

#### Trichosteresis glabra

(Boheman, 1832)

Ceraphron glaber Boheman, 1832
Ceraphron syrphii (Bouché, 1834, *Ceraphron*)
Ceraphron clandestinus (Nees, 1834, *Ceraphron*)
Ceraphron albipennis (Zetterstedt, 1840, *Ceraphron*)
Ceraphron tortricum (Ratzeburg, 1844, *Ceraphron*)
Thliboneura nitida (Thomson, 1858, *Thliboneura*)
Thliboneura radialis (Thomson, 1858, *Thliboneura*)
Trichosteresis floridanus Ashmead, 1887
Trichosteresis foersteri Kieffer, 1907
Trichosteresis flavitarsis Kieffer, 1907
Trichosteresis longigena Kieffer, 1907
Trichosteresis vitripennis Whittaker, 1930
Trichosteresis ninomiyai Yasumatsu, 1963

##### Distribution

England

#### Trichosteresis nudipennis

Kieffer, 1907

## Figures and Tables

**Figure 1a.**
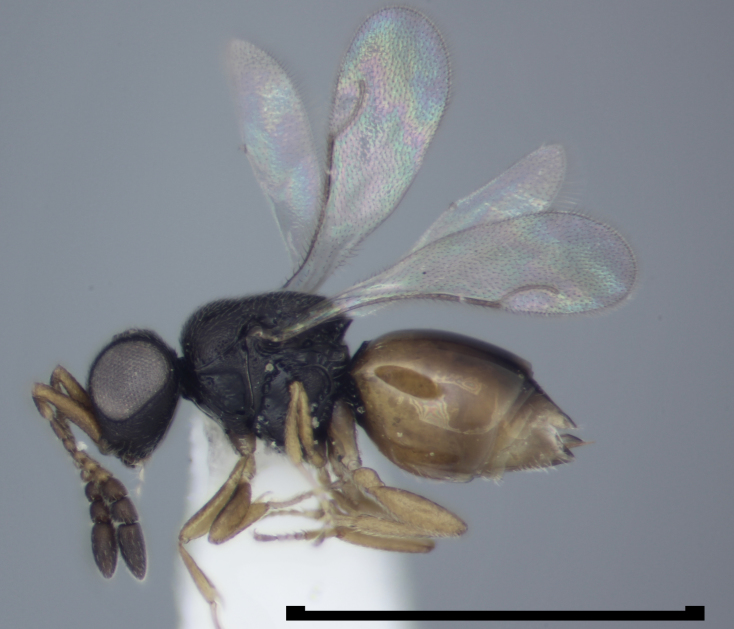
Ceraphronidae: *Aphanogmus abdominalis* (Thomson) female, ex galls of *Dasineura odoratae* Stelter (Diptera: Cecidomyiidae) on *Viola odorata*, England, Surrey, Ripley, July 2007, coll. K.M. Harris, det. P. Buhl (BMNH(E)#1022373).

**Figure 1b.**
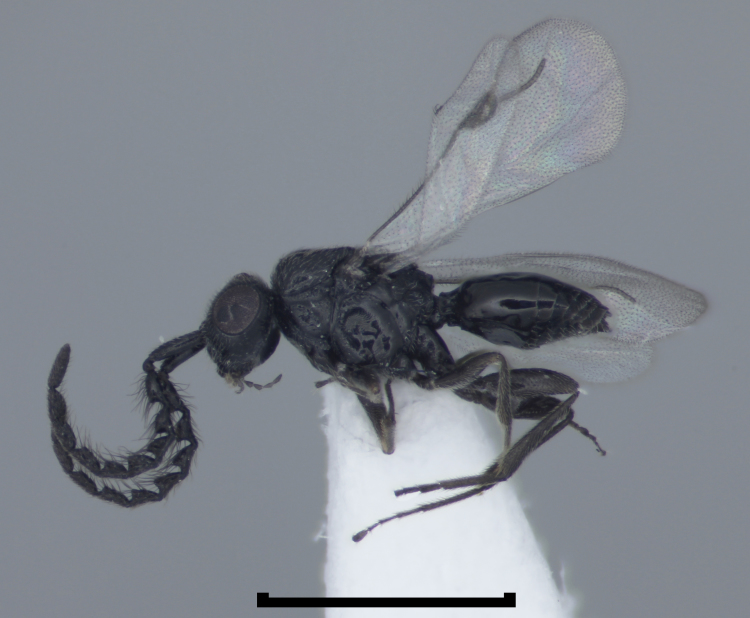
Megaspilidae: Megaspilinae: *Dendrocerus carpenteri* (Curtis) male, ex *Trioxys betulae* Marshall (Hymenoptera: Braconidae) in *Clethrobius comes* (Walker) (Hemiptera: Aphididae) on *Alnus glutinosa*, Scotland, Invernesshire, Dundreggan, June 2012, coll. E.A. Baker, det. D.G. Notton (BMNH(E)#1022374).

**Figure 1c.**
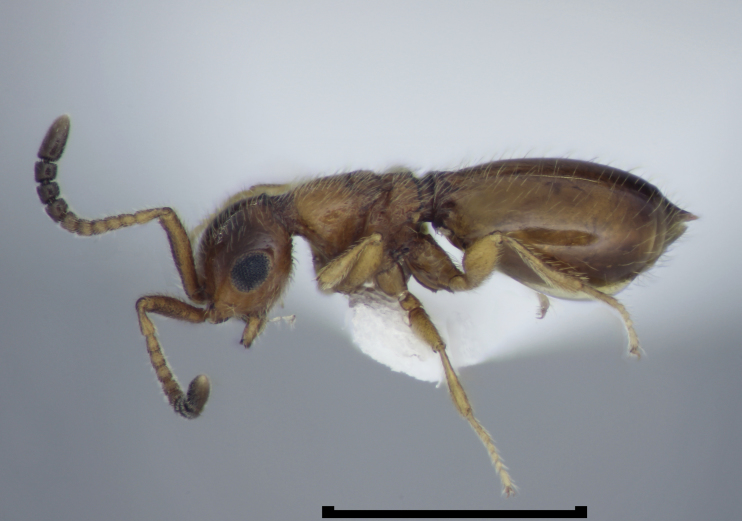
Megaspilidae: Lagynodinae: *Lagynodes pallidus* (Boheman) female, Winkler extraction, England, Isle of Wight, Timber Copse, May 2013, coll. V. Burton, det. D.G. Notton (BMNH(E)#1022375).

**Figure 1d.**
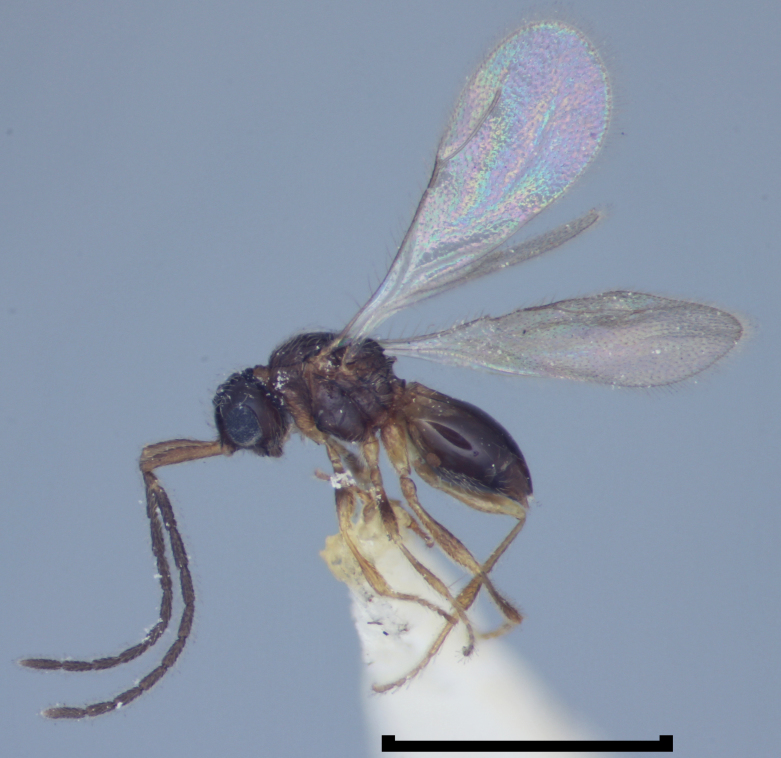
Megaspilidae: Lagynodinae: *Lagynodes pallidus* (Boheman) male, England, Devon, Torquay District, August 1929, coll. G. Nixon (BMNH(E)#1022381).
